# Supplementary material for: Locally Secreted Semaphorin 4D Is Engaged in Both Pathogenic Bone Resorption and Retarded Bone Regeneration in a Ligature-Induced Mouse Model of Periodontitis
Source: Int J Mol Sci. 2022 May 18;23(10):5630. doi: 10.3390/ijms23105630 (PMC9148012; doi:10.3390/ijms23105630)
Supplement: Supplementary file 1 [file ijms-23-05630-s001.zip › ijms-1692142-supplementary.pdf]

Supplementary Figure S1

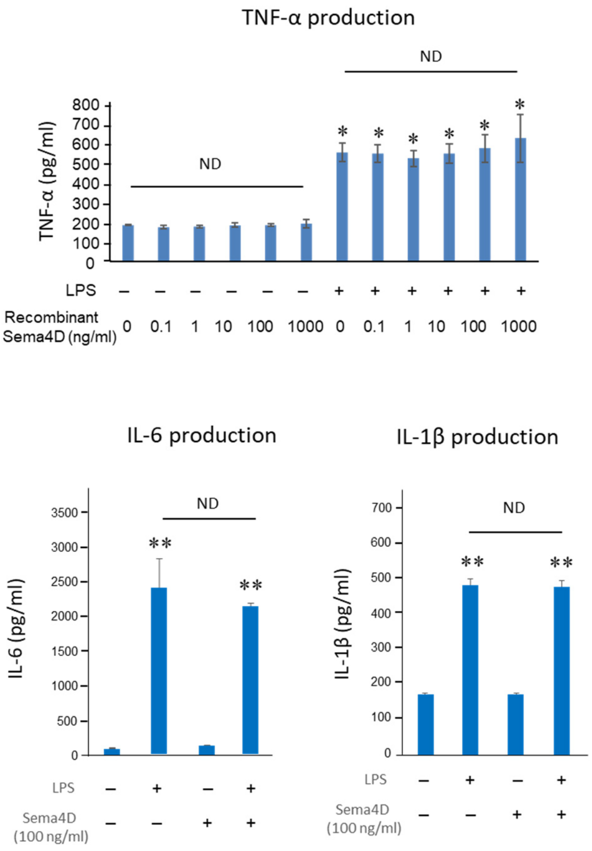

Supplementary Figure: The effect of sSema4D on the proinflammatory response by the mouse bone marrow cells stimulated with LPS.

To examine the effect of sSema4D on the inflammatory response by immune cells, mouse bone marrow cells (10^6 cells/well in a 24 well plate) were stimulated with or without *E. coli* LPS (100 ng/ml) in the presence or absence of recombinant sSema4D (sSema4D-Fc, R&D Systems). After incubation for 24 hours, the culture supernatant was harvested and subeject to ELISA for TNF-α, IL-6 and IL-1β (R&D Systems). Values are means ± S.D. n=3/group **P* < 0.05, ***P* < 0.01 *vs.* BMMCs cultured without LPS or sSema4D, unless indicated by the dotted line. N.D. = no difference between indicated groups.
